# Molecular profiling of the Basal-like intrinsic molecular subtype in primary ER-positive HER2-negative breast cancer

**DOI:** 10.1186/s13073-025-01576-9

**Published:** 2025-12-01

**Authors:** Lennart Hohmann, Deborah F. Nacer, Mattias Aine, Yasin Memari, Daniella Black, Ramsay Bowden, Helen R. Davies, Åke Borg, Johan Vallon-Christersson, Serena Nik-Zainal, Johan Staaf

**Affiliations:** 1 Division of Oncology, Department of Clinical Sciences Lund, Lund University, Lund, Sweden; 2 Division of Translational Cancer Research, Department of Laboratory Medicine, Lund University, Lund, Sweden; 3 Academic Department of Medical Genetics, School of Clinical Medicine & Early Cancer Institute, University of Cambridge, Cambridge, UK

**Keywords:** ER-positive/HER2-negative breast cancer, High-risk breast cancer, PAM50 subtyping, Gene expression profiling, Basal phenotype, Homologous recombination deficiency, DNA methylation profile

## Abstract

**Background:**

The clinical management of ER-positive/HER2-negative (ERpHER2n) breast cancer is complicated by a heterogeneous patient population, with some patients exhibiting endocrine resistance and an increased risk of recurrence. Among these high-risk subgroups, ERpHER2n Basal-like (ERpHER2n-Basal) breast cancer, as defined by PAM50 gene expression subtyping, remains poorly characterized due to limited available material. However, understanding the somatic molecular features driving treatment resistance and progression is critical for optimizing therapy.

**Methods:**

To address these challenges, we comprehensively characterized the patient subgroup by comparing it to both ERpHER2n and triple-negative breast cancer (TNBC) patients. We investigated 4474 Swedish patients with primary ERpHER2n tumors (Basal-like = 76, Luminal A = 3049, Luminal B = 1349) with clinical and RNA-sequencing data available, including 16 Basal-like tumors with whole-genome sequencing and matched global DNA methylation data. For TNBC comparisons, we used an additional 228 cases with available WGS, RNA-sequencing, and DNA methylation data. ER-positivity was defined as ≥ 10% of tumor cells being IHC-stained according to Swedish national guidelines.

**Results:**

Clinicopathological analyses highlighted ERpHER2n-Basal patients as a small subgroup comprising generally younger patients with high-grade and high-risk tumors. This patient group was associated with worse prognosis than Luminal A/Luminal B subtypes, especially when treated only with endocrine therapy, independent of lymph node status, patient age, tumor size and grade. Molecularly, ERpHER2n-Basal tumors were distinguished by high proliferation and elevated immune response together with low *ESR1* mRNA expression and low activity of steroid-response pathways. High proportions of the mutational signatures associated with homologous recombination deficiency in ERpHER2n-Basal tumors suggest potential benefits from platinum or PARP inhibitor treatments. Additionally, their DNA methylation profile closely resembles that of Basal triple-negative breast cancer (TNBC), indicating shared epigenetic regulation despite differences in ER status. Further molecular similarities to TNBC such as high immune infiltration indicate immune checkpoint inhibitors as promising agents for improving patient care.

**Conclusions:**

ERpHER2n-Basal breast cancer represents a clinically high-risk subgroup whose molecular resemblance to TNBC highlights potential therapeutic opportunities, particularly for immunotherapy and DNA repair–targeting treatments.

**Supplementary Information:**

The online version contains supplementary material available at 10.1186/s13073-025-01576-9.

## Background

Breast cancer is a heterogeneous disease classified into clinical subgroups based on the assessment of three main treatment predictive biomarkers: protein overexpression of the estrogen receptor (ER) and progesterone (PR) receptor, and overexpression/gene amplification of the human epidermal growth factor receptor 2 (*ERBB2/HER2*) gene. ER-positive HER2-negative (ERpHER2n) tumors represent the largest group (approximately 70–80%) of breast cancer and are also collectively referred to as luminal [1]. Beyond clinical markers, gene expression profiling has refined breast cancer classification, with the PAM50 signature identifying five intrinsic subtypes: Luminal A (LumA), Luminal B (LumB), HER2-enriched, Basal-like, and Normal-like [2, 3].

Unlike the luminal subtypes LumA and LumB, PAM50 Basal-like tumors share features with triple-negative breast cancer (TNBC), though the two are not entirely synonymous. Approximately 70–80% of TNBC tumors are classified as Basal-like and are particularly aggressive, displaying elevated proliferation and heightened immune activation [4]. However, Basal-like tumors also occur outside the TNBC group. When applied to population-representative breast cancer cohorts the dominant PAM50 subtypes in ERpHER2n tumors are LumA and LumB, yet small subgroups of both Basal-like and HER2-enriched tumors are also observed [5, 6].

ERpHER2n tumors classified as Basal-like (ERpHER2n-Basal) demonstrate a distinct molecular and clinical behavior that challenges conventional treatment paradigms based on ER status. Despite their clinical classification as ER-positive, these tumors respond poorly to endocrine therapy and exhibit resistance to CDK4/6 inhibition [7], aligning with features of estrogen-independence [8] and indicating a need for alternative therapeutic strategies. Given the success of immune checkpoint inhibitors and PARP inhibitors in TNBC [9], investigating whether ERpHER2n-Basal tumors could benefit from similar treatment approaches is promising. However, their rarity has led to limited representation in clinical trials, restricting the evidence needed to guide treatment decisions. A deeper molecular characterization of this subgroup is therefore needed to refine patient selection for targeted treatment approaches in luminal disease and to identify features that could serve as potential new therapeutic targets to improve outcomes.

In the current study we aimed to comprehensively characterize ERpHER2n-Basal breast cancer through comparison to other ERpHER2n subgroups, as well as to TNBC, based on clinicopathological and molecular features on the RNA, DNA, and epigenetic level. We evaluate the response of patients to conventionally administered therapies and identify features with the potential to inform treatment decisions. A key aspect of the study is the primary focus on ER-positive tumors defined by ≥ 10% positively stained tumor cells, thus excluding the proposed ER-low subgroup of tumors with 1–9% ER staining [10]. The key finding of this study is that ERpHER2n-Basal tumors are molecularly distinct from luminal subgroups yet share striking biological similarities with TNBC Basal-like tumors. This suggests that, despite differences in clinical ER classification, ERpHER2n Basal-like tumors given their specific molecular characteristics may benefit from similar treatment strategies used in TNBC.

## Methods

### Patient cohorts

The analyses in this study were based on early-stage breast cancer data from the Sweden Cancerome Analysis Network – Breast (SCAN-B) study reported by Staaf et al. [5] and validated using the Molecular Taxonomy of Breast Cancer International Consortium (METABRIC) study [11] as an independent cohort. To contrast whole genome sequencing (WGS) data for ERpHER2n-Basal SCAN-B tumors in ERpHER2n disease, the cohort reported by Nik-Zainal et al. was used, hereafter referred to as BASIS [12]. To contrast DNA methylation patterns for ERpHER2n-Basal SCAN-B tumors versus TNBC, the SCAN-B TNBC cohort reported by Aine et al. was used [13]. To contrast DNA methylation patterns for ERpHER2n-Basal SCAN-B tumors in breast cancer, irrespective of ER, PR and HER2 status, 645 tumors from the TCGA study were used (https://www.cancer.gov/tcga). Patient inclusion and exclusion criteria for the used cohorts are available from original publications [1, 5, 11–14]. In all cohorts, the PAM50 subtype characterization was obtained from publicly deposited data, or as outlined in the original study, and used to form the class labels for the subsequent analyses. In all cohorts and analyses, a tumor sample is represented by a single analysis of respective type (e.g., WGS analysis, RNA-sequencing, DNA methylation analysis). In contrast to our primary SCAN-B discovery cohort, comprehensive population representativity analyses for the other included cohorts have not been reported. This represents a potential limitation specifically of the validation cohort METABRIC, which includes patients diagnosed between 1977 and 2005, with respect to conclusions about the generalizability of results derived from this cohort alone.

#### SCAN-B

Cases from the population-representative early-stage SCAN-B cohort recently reported by Staaf et al. [5] constituted the focal point of analyses included in the clinicopathological and molecular characterization of the ERpHER2n-Basal subtype. The SCAN-B study (ClinicalTrials.gov ID NCT02306096) [1, 15] is a prospective, observational, cohort study. The SCAN-B study is approved by the Regional Ethical Review Board in Lund, Sweden (registration numbers 2009/658, 2010/383, 2012/58, 2013/459, 2014/521, 2015/277, 2016/541, 2016/742, 2016/944, 2018/267) and the Swedish Ethical Review Authority (registration numbers 2019–01252, 2024–02040-02). All patients provided written informed consent prior to enrolment. All analyses were performed in accordance with patient consent and ethical regulations and decisions.

From this cohort, we extracted clinical cancer registry data and RNA-sequencing data from 4474 ERpHER2n cases. Of these 4474 tumors, 76 (2%) were classified as Basal-like, 3049 (68%) as LumA, and 1349 (30%) as LumB according to available PAM50 nearest centroid classification. Expression data for 19309 genes was available as FPKM (Fragments Per Kilobase per Million mapped fragments) estimates from the online repository associated with the study [5]. To target global DNA and epigenetic alterations, WGS was performed for 16 of the 76 ERpHER2n-Basal tumors. Tumors were selected based on accessibility to DNA from patients diagnosed between 2010 and 2014 in the Skåne healthcare region in South Sweden. Matched global DNA methylation profiles based on Illumina EPIC v1.0 beadchips for all WGS analyzed tumors were obtained from Hohmann et al. [16] (available on Gene Expression Omnibus, GEO, under accession number GSE278586). WGS and DNA methylation analyses were performed using the same starting DNA for each tumor. These ERpHER2n SCAN-B cases were used to compare ERpHER2n-Basal tumors with LumA and LumB tumors based on clinicopathological characteristics, survival outcomes, and transcriptional profiles. Transcriptomic analyses included mRNA expression of selected genes and metagenes, UMAP clustering, differential gene expression, and gene ontology enrichment analysis.

For the comparison of SCAN-B ERpHER2n-Basal tumors to TNBC, processed WGS, RNA-sequencing (FPKM), and DNA methylation data (beta values, Illumina EPIC v1.0 beadchips) from 228 SCAN-B TNBC cases classified as eligible for follow-up analyses were obtained from public repositories associated with the studies by Staaf et al. [5, 17] and Aine et al. [13] (GEO, accession numbers GSE148748 and GSE148906). The two contrasting groups within TNBC were defined as cases that were PAM50 subtyped as Basal-like (TNBC-Basal) or another subtype (TNBC-NonBasal). Of the 228 TNBC cases, 184 (81%) were classified as TNBC-Basal and 44 (19%) as TNBC-NonBasal, which were used in comparative analyses to ERpHER2n-Basal cases that included mRNA expression of selected genes and metagenes, UMAP clustering, differential gene expression, and gene ontology enrichment. The validation of immune infiltration was based on pathology assessed tumor infiltrating lymphocyte (TIL) counts that were obtained from Aine et al. [18] for 207 of the used TNBC tumors. Further genomic analyses included assessments of driver alterations, HRD status, and autosomal genome-wide copy number alterations. For comparison of DNA methylation patterns to the WGS-profiled ERpHER2n-Basal cases (*n* = 16), the TNBC cohort was epigenetically stratified into epiBasal (*n* = 172) and epiNonBasal (*n* = 56) subgroups based on the study by Aine et al. [13]. The ERpHER2n-Basal tumors were subsequently co-analyzed with both subgroups to evaluate similarity in DNA methylation profiles. Additionally, promoter region methylation maps for *FOXA1* and *FOXC1* were generated to assess the correlation between observed transcriptional and epigenetic alterations.

#### BASIS

We obtained WGS data for 178 ERpHER2n cases, including 73 LumA and 105 LumB classified tumors, from the study by Nik-Zainal et al. [12]. These cases were used to contrast the genomic profiles of the WGS-profiled ERpHER2n-Basal cases (*n* = 16) with ERpHER2n LumA and LumB tumors, including analyses of driver alterations, mutational burden, and mutational signatures. In addition, the BASIS tumors were used for comparing the frequency of HRD-positivity and to evaluate autosomal copy number alteration frequencies across the genome.

#### TCGA

Matched DNA methylation (Illumina 450 K beadchips) and RNA-sequencing data for 645 TCGA tumors representing all clinical subgroups were obtained as described by Staaf and Aine [19]. TCGA tumors were PAM50 subtyped using the extended nearest centroid approach described for SCAN-B tumors by Staaf et al., using the same reference sets and code [5]. TCGA tumors were co-analyzed with ERpHER2n-Basal tumors to contrast their DNA methylation patterns versus all clinical subgroups of breast cancer and to compare promoter methylation patterns of *FOXA1* and *FOXC1*.

#### METABRIC

The METABRIC validation cohort provided clinical, mutational (targeted NGS), transcriptomic (Illumina HT-12 v3 platform), and copy number (Affymetrix SNP 6.0 platform) data for 1075 ERpHER2n cases with available PAM50 class [11, 20]. Of the 1075 cases, 44 (4%) were PAM50 subtyped as Basal-like, 635 (59%) as LumA, and 396 (37%) as LumB. For comparisons to TNBC, METABRIC provided data on 243 TNBC tumors, of which 209 (86%) were classified as TNBC-Basal, and 34 (14%) as TNBC-NonBasal. METABRIC data was downloaded from the cBioPortal website (https://www.cbioportal.org/) as pre-compiled data. Expression data for 24360 genes were available as z-score estimates. These cases were used to validate the main observations in the SCAN-B cohort regarding mRNA expression patterns of genes and metagenes, as well as the driver gene alterations landscape in ERpHER2n-Basal tumors.

#### ER, PR, and HER2-status determination

In the METABRIC cohort, the HER2-status was determined by using SNP 6.0 copy number array data for the *ERBB2* gene, whereas in BASIS it was verified from WGS data for *ERBB2*. In the SCAN-B cohort HER2-status was determined from routine clinical IHC and FISH analysis as reported in the Swedish national breast cancer quality registry and assessed according to the standardized protocols described in the KVAST guidelines of the Swedish Society of Pathologists [21]. Briefly, a HER2n tumor in Sweden is defined by an IHC HER2-staining score of < 2, or for patients with IHC 2 +, a non-amplified ISH status. In SCAN-B, ER and PR data is based on routine clinical IHC analysis as reported in the national cancer quality registry. Specifically, ER-positivity was defined as ≥ 10% of tumor cells being IHC-stained according to current Swedish national guidelines. In the METABRIC cohort, ER-positivity is expected to be defined as ≥ 1% IHC-stained tumor cells. Due to specific ER IHC scores not being available, reclassifying METABRIC cases according to Swedish national guidelines was not possible.

### Statistics and survival analyses

Clinicopathological variables were compared using Fisher's exact or Mann–Whitney U tests, with two-sided p-values reported. Survival analyses utilized the survival (v3.4.0) [22] and survminer (v0.4.9) [23] packages. Kaplan–Meier survival curves were compared using log-rank tests, and hazard ratios were calculated using Cox regression. Clinical endpoints included distant recurrence-free interval (DRFI) and overall survival (OS). Multivariate Cox models incorporated age, lymph node status, tumor size, and grade as covariates to PAM50 subtypes. Patients were grouped according to treatment into endocrine therapy (ET) or combined chemotherapy and endocrine therapy (CT + ET) cohorts. All data processing and statistical analyses were performed using the R programming language (v4.2.2) [24]. Statistical methods are further detailed in Additional File 1: Supplementary Methods**.**

### Gene expression analyses

The transcriptomic characterization of ERpHER2n-Basal tumors in ERpHER2n breast cancer involved analyses of selected genes and metagenes, supervised differential gene expression, gene set enrichment, unsupervised clustering, and PAM50 centroid correlation comparisons as described in Hohmann et al. [16] and as detailed in Additional File 1: Supplementary Methods. The GO enrichment analysis for biological processes was performed using the R clusterProfiler (v4.12.6) package [25], with gene annotations sourced from the org.Hs.eg.db database [25].

### Analysis of genomic mutational patterns

Analyses of single nucleotide variants (SNVs), indels, and rearrangements in the 16 WGS analyzed ERpHER2n-Basal SCAN-B tumors, were performed and contrasted against cases of the BASIS cohort as described in Hohmann et al. [16]. Mutational signatures for single-base substitutions (SBSs) and structural rearrangements were identified following established methods [26] and homologous recombination deficiency (HRD) was assessed using HRDetect [27]. Additional details on the methods used for these analyses are provided in Additional File 1: Supplementary Methods. In the METABRIC validation cohort, mutational analyses were based on pre-processed targeted NGS data and limited to the assessment of mutational frequencies and landscape of driver alterations.

### Copy number alteration analyses

Copy number analyses were performed using copy number estimates obtained from WGS (*n* = 16 ERpHER2n-Basal tumors) which were processed as described in Staaf et al. [17] and Additional File 1: Supplementary Methods, including allele-specific segmentation using ASCAT [28] and calling of copy number alterations versus tumor ploidy. Further details are found in Additional File 1: Supplementary Methods.

### DNA methylation analysis

Matched global DNA methylation profiles based on Illumina EPIC ver1 beadchips for WGS analyzed ERpHER2n-Basal tumors were obtained from Hohmann et al. [16] (GSE278586). Methylation profiling was performed by the SNP&SEQ Technology Platform in Uppsala (www.genotyping.se). Preprocessing of DNA methylation beta values from GSE278586, representing the level of methylation, and adjustment of beta values for tumor purity using the PureBeta pipeline [29] were performed as outlined in Additional File 1: Supplementary Methods. Matched DNA methylation (Illumina 450 K beadchips) data for 645 TCGA tumors were obtained as described by Staaf and Aine [19] and also corrected for tumor purity as described in the same study.

## Results

**Fig. 1:**
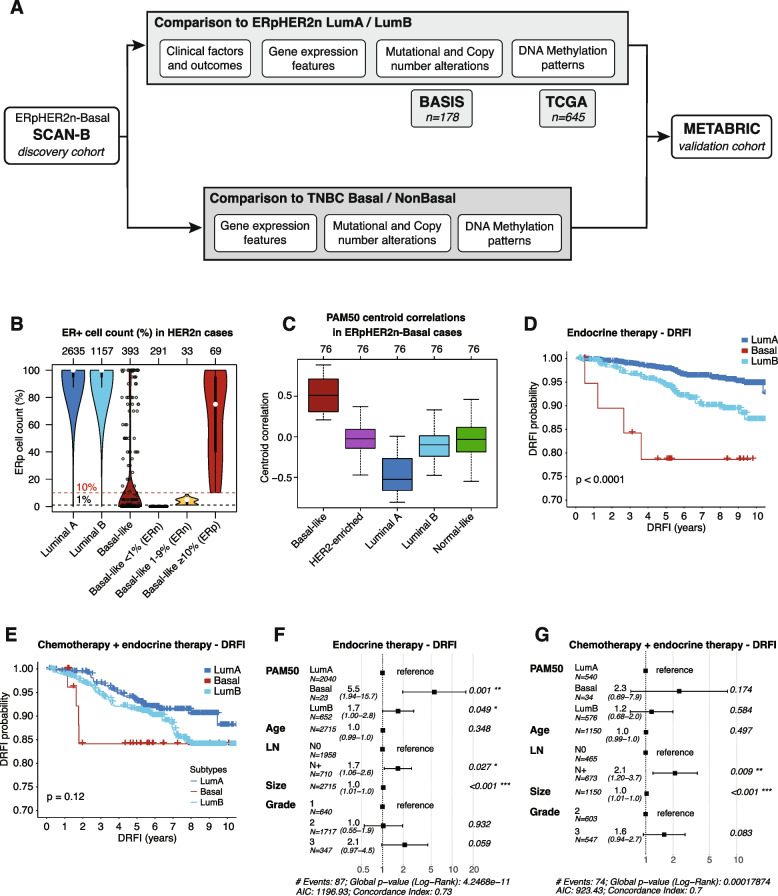
Study overview and survival analyses of PAM50 subtypes in ERpHER2n SCAN-B patients with DRFI as clinical endpoint. **A** Study overview. **B** Percentage of ER-positive cells in HER2-negative tumors by PAM50 subtypes (Basal further stratified by ER-status definition cut-offs). **C** PAM50 centroid correlations in ERpHER2n-Basal cases. **D** Kaplan–Meier curve of patients treated with endocrine therapy (ET) stratified by PAM50 status using distant recurrence-free interval (DRFI) as endpoint. **E** Kaplan–Meier curve of patients treated with chemotherapy and endocrine therapy (CT + ET) stratified by PAM50 status using DRFI as endpoint. **F** Multivariate Cox regression hazard ratios (HRs) with 95% confidence intervals (CIs) for patients treated with ET. **G** Multivariate Cox regression HRs with 95% CIs for patients treated with CT + ET. *Differences in Kaplan–Meier curves: p-values calculated using log-rank tests. For multivariate cox regression in the CT* + ET group, grade 1 tumors had to be excluded due to no associated events. Top axes in plots report group sizes. LN: lymph node status. Significance annotation: * ≤ 0.05; ** ≤ 0.01; *** ≤ *0.001*

The current study aimed to perform an in-depth clinicopathological and molecular characterization of ERpHER2n-Basal tumors based on comparisons with ERpHER2n LumA and LumB tumors and with TNBC tumors. Figure 1A shows an outline of the performed analyses and cohorts used.

### ERpHER2n-Basal disease and clinicopathological variables

In the ERpHER2n SCAN-B cohort (*n* = 4474) 2% of tumors (*n* = 76) were classified as Basal-like. Analyses of clinicopathological variables for patients with ERpHER2n-Basal tumors versus patients with LumA and LumB tumors demonstrated that they occur at a younger age, are more often PR negative and of higher grade. The 21-gene Recurrence Score (RS) classification, based on RNA-sequencing FPKM data, identified all ERpHER2n-Basal tumors as high-risk. Similarly, the Risk of Recurrence (ROR) score classification [5] categorized 70% of ERpHER2n-Basal tumors as high-risk. Taken together, these assessments show that ERpHER2n-Basal patients have a risk level comparable to LumB patients and significantly higher than LumA patients (Table 1).

**Table 1:** Clinicopathological characteristics of the intrinsic molecular subtypes in ERpHER2n breast cancer

	**Basal (ref)**	**LumA**	**LumB**
Number of samples	76 (2%)	3049 (68%)	1349 (30%)
Tumor size (mm)	20.1 ± 13.5	18.4 ± 12.1	21.6 ± 12.3
Tumor grade			
NHG1	10 (14%)	834 (28%)***	73 (6%)***
NHG2	18 (26%)	1929 (64%)***	593 (45%)***
NHG3	42 (60%)	245 (8%)***	640 (49%)***
Lymph node status			
N0	53 (73%)	1994 (67%)	802 (60%)*
N +	20 (27%)	1001 (33%)	526 (40%)*
Patient age (years)	60.8 ± 13.9	65.3 ± 12.2**	67.5 ± 12.8***
PR positive	35 (46%)	2701 (89%)***	1076 (80%)***
21-gene RS^Subset^	87.7 ± 16.6	24.7 ± 14.4***	57.3 ± 31.1***
21-gene RS risk group^Subset^			
Low	0 (0%)	990 (33%)***	159 (12%)***
Intermediate	0 (0%)	1062 (36%)***	162 (12%)***
High	70 (100%)	927 (31%)***	999 (76%)***
ROR score^Subset^	62.9 ± 16.7	27.7 ± 14.3***	67.0 ± 10.8
ROR risk group^Subset^			
Low	6 (9%)	1751 (59%)***	0 (0%)***
Intermediate	15 (21%)	816 (27%)***	271 (21%)***
High	49 (70%)	412 (14%)***	1049 (79%)***

Percentages were calculated across subtypes for number of samples and within subtypes for the other variables. Risk group classifications were performed only for a subset of cases with data available in the study by Staaf et al. [5]. Statistics: Fisher’s exact tests for variables PR, Tumor grade, lymph node status, 21-gene Recurrence Score (RS) risk groups and Risk of Recurrence (ROR) risk groups; Mann–Whitney U tests for variables Patient age, Tumor size, RS and ROR score. Pairwise comparisons were made between Basal and LumA or Basal and LumB

Significance annotation: * ≤ 0.05; ** ≤ 0.01; *** ≤ 0.001

Figure 1B shows available clinical ER IHC scores for HER2-negative tumors which were PAM50 classified as LumA, LumB, or Basal-like and then further demonstrates how Basal-like tumors are classified regarding ER-positivity according to the cut-offs recommended in Swedish (≥ 10%) and international (≥ 1%) guidelines [30]. Notably, the ER-positive Basal-like group is lower in ER scores than LumA and LumB tumors, but still by definition markedly higher on a group level compared to TNBC cases (both according to Swedish and international ER-positivity cut-offs). Consistent with their PAM50 subtype, ERpHER2n-Basal tumors exhibited the highest correlation to the PAM50 Basal-like centroid, while markedly lower to other PAM50 centroids (Fig. 1C). The latter suggests a general distinctness of the ERpHER2n-Basal tumor’s transcriptional profile.

### ERpHER2n-Basal tumors and associations with patient outcome

To assess the association of the ERpHER2n-Basal tumor phenotype with patient outcome we performed survival analysis stratified by treatment (endocrine only, ET, or combined chemotherapy and endocrine therapy, CT + ET) using distant recurrence-free interval (DRFI) and overall survival (OS) as clinical endpoints. For both treatment groups, patients with ERpHER2n-Basal tumors showed a trend towards early recurrences (Fig. 1D-E). In patients treated with adjuvant ET, ERpHER2n-Basal patients showed a significantly poorer DRFI than both LumA and LumB patients (Fig. 1D). This observation was supported by a multivariate Cox regression analysis, which demonstrated that the ERpHER2n-Basal subtype provided independent prognostic information in a model with patient age, lymph node status, tumor size and tumor grade as covariates (Hazard ratio (HR) = 5.5, 95% confidence interval (CI) = 1.94–15.7) (Fig. 1F). Consistently, poor outcomes in ET-treated ERpHER2n-Basal patients were also observed using OS as clinical endpoint, with ERpHER2n-Basal patients showing a significantly worse survival compared to LumA patients (multivariate Cox regression HR = 2.0, 95% CI = 1.03–4.0, *p* = 0.04).

In the CT + ET group, patients with ERpHER2n-Basal tumors did not have a statistically significantly poorer DRFI compared to LumA and LumB patients, despite a different relapse pattern (Fig. 1E). Consistently, the ERpHER2n-Basal subtype did not add independent prognostic information in multivariate Cox regression analysis (Fig. 1G). With respect to OS, the association between the ERpHER2n-Basal subtype and poor OS was significant in univariate Cox regression in the CT + ET group (HR = 2.6, 95% CI = 1.1–6.0, *p* = 0.031). However, after adjusting for other variables in the multivariate analysis, the association was no longer statistically significant, although a trend remained (multivariate Cox regression HR = 2.5, 95% CI = 0.88–7.3, *p* = 0.085) (Additional File 1: Fig. S1).

### Transcriptional patterns of ERpHER2n-Basal disease

**Fig. 2:**
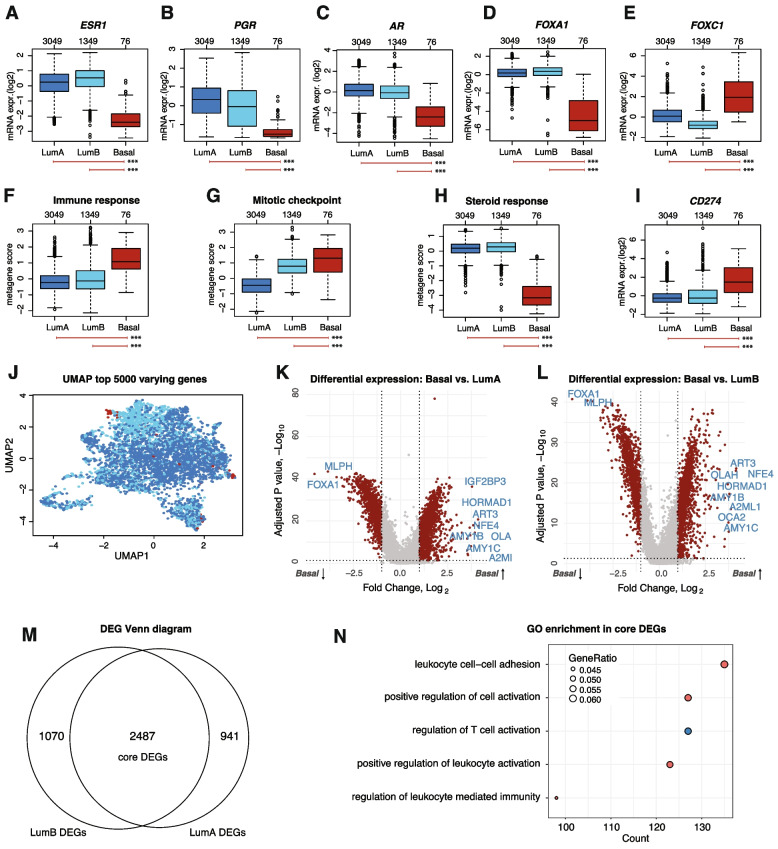
Transcriptomic analyses in ERpHER2n SCAN-B tumors. **A**-**E** Scaled mRNA expression of *ESR1, PGR, AR, FOXA1* and *FOXC1.* **F**–**H** Rank scores of the immune response, mitotic checkpoint (proliferation), and steroid response metagenes. **I** Scaled mRNA expression of *CD274* (*PD-L1*). **J** UMAP analysis including all samples based on FPKM expression data of the top 5000 varying genes. Colors represent PAM50 subtypes as in previous panels. **K** Volcano plot of DEGs between the Basal and LumA subtypes. **L** Volcano plot of DEGs between the Basal and LumB subtypes. **M** Venn diagram of the overlap of differentially expressed genes (DEGs) between Basal vs LumA and Basal vs LumB tumors. **N** GO enrichment analysis for biological processes of core DEGs, where *GeneRatio* indicates the proportion of genes in the dataset linked to each GO term and *Count* the total number of genes associated with each term. In panels including boxplots, two-sided p-values were computed using Mann–Whitney's test, top axes report group sizes. Significance annotation: * ≤ 0.05; ** ≤ 0.01; *** ≤ *0.001. Boxplot elements correspond to: (i) center line* = *median, (ii) box limits* = *upper and lower quartiles, (iii) whiskers* = *1.5* × *interquartile range*

Focusing on genes associated with ER and steroid response, ERpHER2n-Basal tumors showed significantly lower mRNA expression of *ESR1*, *PGR*, *AR*, and *FOXA1*, consistent with the lower ER IHC scores and higher frequency of PR negativity (Fig. 2A-D). In contrast, *FOXC1* (a PAM50 gene) showed significantly higher mRNA expression in ERpHER2n-Basal tumors, consistent with its reported expression profile in Basal-like TNBC [31] (Fig. 2E). Using previously reported biological gene expression metagenes in breast cancer [32], ERpHER2n-Basal tumors showed significantly higher rank scores of the immune response and proliferation metagenes, while lower rank scores of the steroid response metagene compared to LumA and LumB tumors (Fig. 2F-H). We also observed a significantly higher mRNA expression of *PD-L1* (*CD274*) in ERpHER2n-Basal tumors compared to LumA and LumB tumors, which may contribute to enhanced immune evasion through the PD-1/PD-L1 immune checkpoint pathway (Fig. 2I). Although immune infiltration estimates based on RNA-sequencing should ideally be validated in situ, for instance through estimation of tumor infiltrating lymphocytes (TILs), previous findings in SCAN-B TNBC tumors have demonstrated that the specific expression metagene in Fig. 2F shows high correlation to TIL counts based on pathologist scoring [33].

To investigate whether ERpHER2n-Basal tumors displayed a more diverse transcriptional profile than other ERpHER2n tumors we performed an unsupervised UMAP analysis of the 4474 SCAN-B tumors using the top 5000 genes most varying in expression, finding that they do not form a completely distinct transcriptional entity (Fig. 2J). Next, we performed supervised differential gene expression between ERpHER2n-Basal versus LumA tumors and ERpHER2n-Basal versus LumB tumors, identifying in each comparison a large number of differentially expressed genes (DEGs) that also showed a large gene overlap (Fig. 2K-M, Additional File 2). A GO enrichment analysis for biological processes identified a significant enrichment of immune response-associated pathways in the core set of 2487 genes differentially expressed in both comparisons, consistent with the elevated expression pattern of the immune response metagene (Fig. 2N).

### Mutational and driver gene characterization of ERpHER2n-Basal disease

**Fig. 3:**
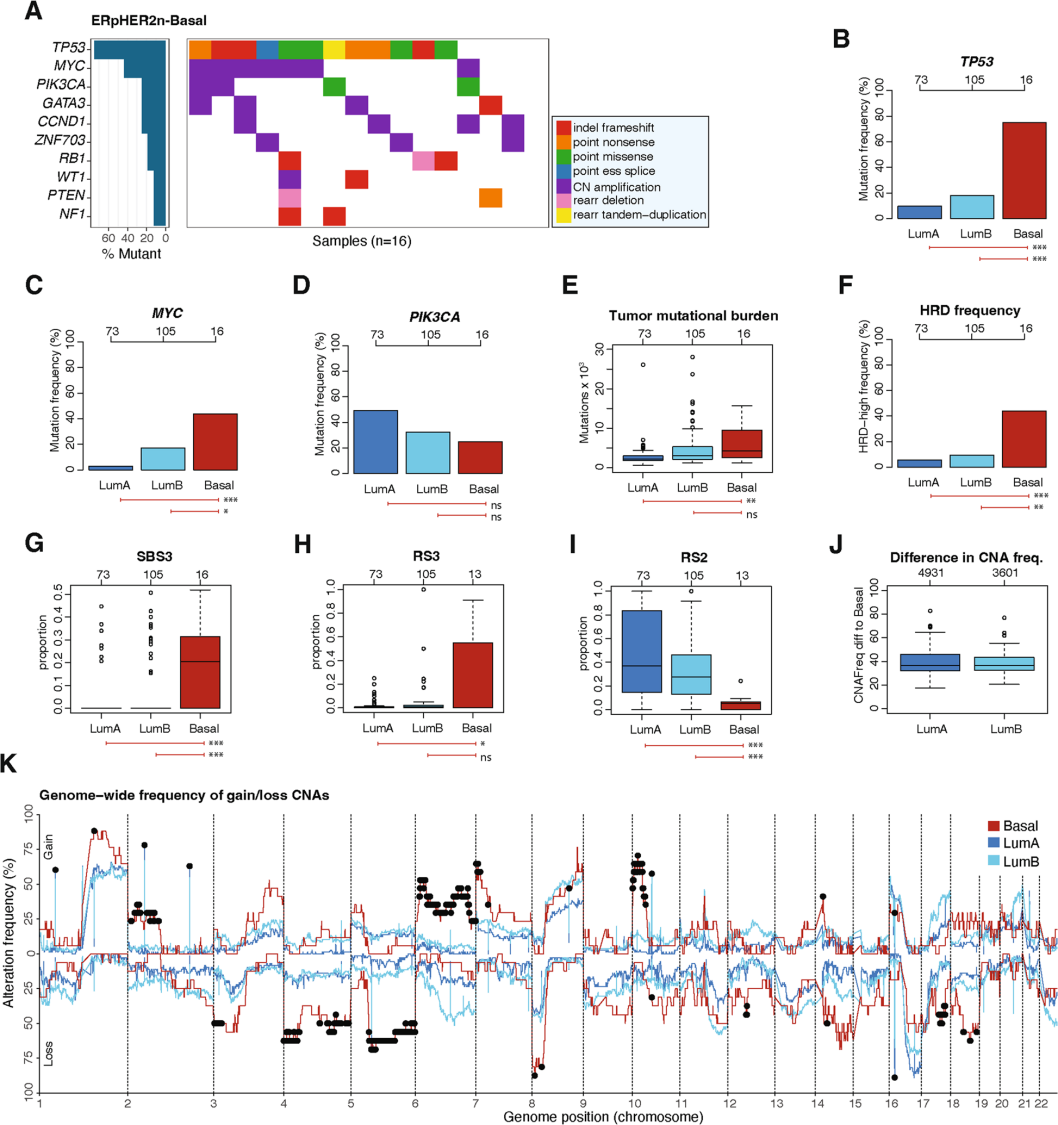
Mutational and copy number alteration landscape of ERpHER2n-Basal tumors.** A** Top ten driver genes with most alterations in Basal tumors. **B**-**D** Comparison of driver alteration frequencies between Basal, LumA, and LumB tumors for *TP53*, *MYC*, and *PIK3CA*. **E** Overall mutational burden across subtypes. **F** Predicted HRD frequency across subtypes using HRDetect. **G-I** Exposure of single base substitution signature 3 (SBS3), rearrangement signature 3 (RS3), and rearrangement signature (RS2) across PAM50 subtypes. **J** Absolute difference in copy number alteration frequencies between Basal and Luminal subtypes for genes with significantly different copy number status between subtypes in pairwise comparisons. **K** Autosomal copy number alteration landscape of Basal tumors. Black dots mark genes that are significant between both Basal-LumA tumors and Basal-LumB tumors. In panels including boxplots, two-sided p-values were computed using Mann–Whitney's test, top axes report group sizes. Significance annotation: * ≤ 0.05; ** ≤ 0.01; *** ≤ *0.001. Boxplot elements correspond to: (i) center line* = *median, (ii) box limits* = *upper and lower quartiles, (iii) whiskers* = *1.5* × *interquartile range*

We characterized the mutational landscape of ERpHER2n-Basal tumors by performing WGS on 16 SCAN-B tumors (Additional File 3) and comparing their mutational profiles to LumA and LumB tumors from the BASIS cohort. While limited by sample numbers, the top five driver genes with alterations in ERpHER2n-Basal tumors were *TP53*, *MYC*, *PIK3CA*, *GATA3*, and *CCND1* (Fig. 3A). Compared to driver alterations in LumA and LumB, the high frequency of *TP53* mutations in ERpHER2n-Basal tumors represented the most significant difference, followed by a higher number of *MYC* amplifications, while *PIK3CA* was less frequently altered (Fig. 3B-D). The overall mutational burden was higher in ERpHER2n-Basal tumors than in LumA tumors but not compared to LumB tumors (Fig. 3E). HRD classification using HRDetect [27] revealed a 44% HRD frequency (*n* = 7/16) in ERpHER2n-Basal tumors, significantly higher than in LumB and LumA tumors (Fig. 3F). Consistent with a frequent HRD phenotype [27], we observed higher exposure to both single base substitution signature 3 and rearrangement signature 3, while lower exposure to rearrangement signature 2 (Fig. 3G-I). Furthermore, we found *HORMAD1* to be differentially overexpressed in ERpHER2n-Basal tumors versus both LumA and LumB tumors (Fig. 2K-L), a gene previously found to contribute to HRD in TNBC [34].

### The ERpHER2n-Basal copy number alteration landscape

Using again the SCAN-B and BASIS WGS-analyzed ERpHER2n tumors, a statistical analysis of copy number gain and loss frequencies for 19996 genes across the genome revealed 4931 genes that differed between the ERpHER2n-Basal and LumA subtypes, and 3601 genes that differed between the ERpHER2n-Basal and LumB subtypes (Additional File 4). For the statistically significant genes, the mean absolute difference in alteration frequency was 37% for LumA and 36% for LumB (Fig. 3J). To visualize the autosomal copy number landscape of ERpHER2n-Basal tumors, we compared their gene gain and loss frequencies against the frequency profiles of BASIS LumA and LumB tumors (Fig. 3K). ERpHER2n-Basal tumors displayed marked differences in copy number loss at chromosomes 3p, 4p, 4q, 5q, 8p, 17q, and 18q, while notable higher frequency of copy number gain at chromosomes 2p, 6p, 6q, 7p, and 10p.

### Molecular features associated with the PAM50 Basal-like subtype are consistent regardless of ER-status

**Fig. 4:**
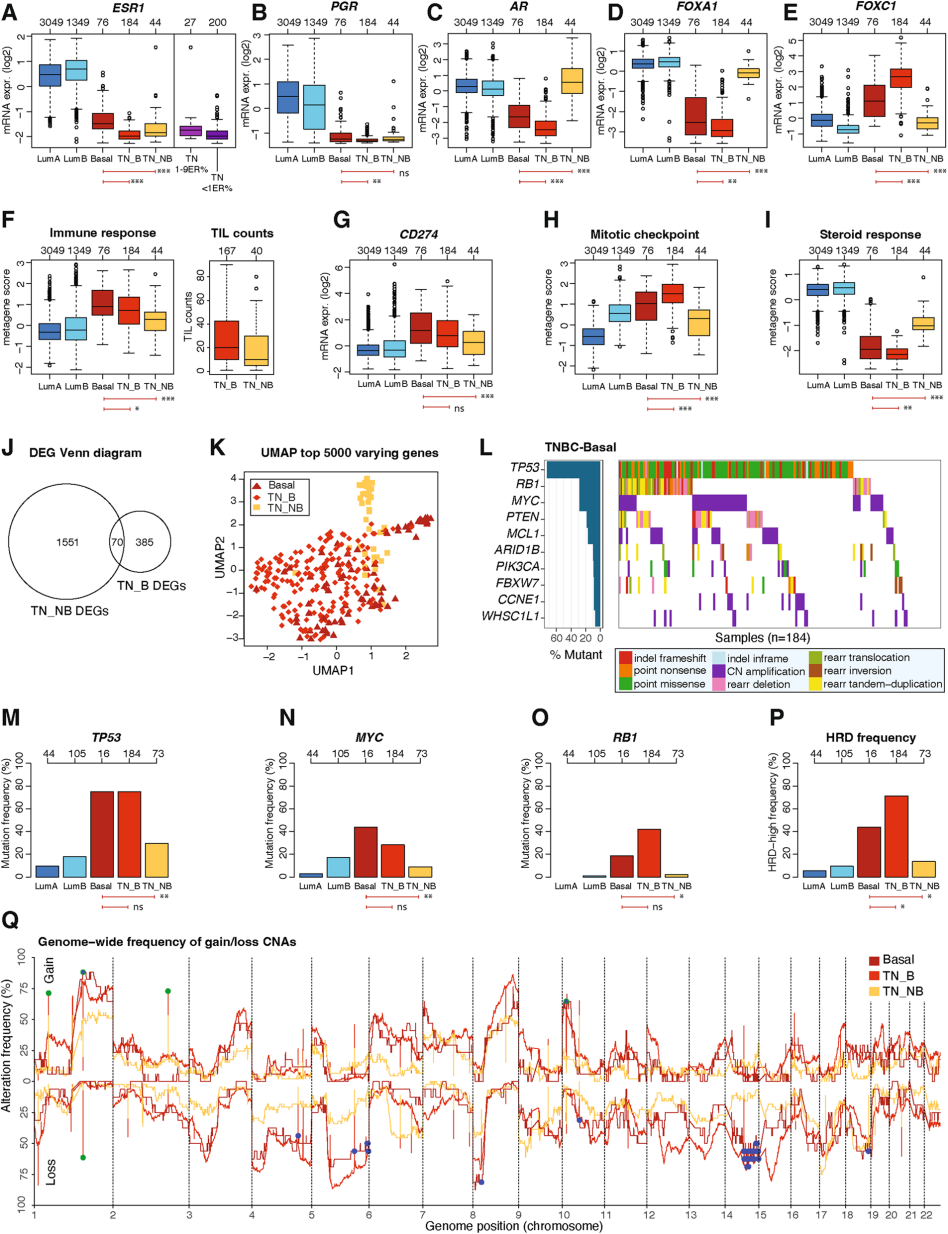
Molecular feature comparisons between ERpHER2n-Basal tumors and TNBC, with LumA and LumB included for biological context**. A** Scaled mRNA expression of *ESR1* for ERpHER2n-Basal tumors compared to TNBC-Basal (TN_B) and TNBC-NonBasal (TN_NB) tumors. Plotted alongside is the *ESR1* mRNA expression of TNBC tumors with ER staining of 1–9% (TN 1-9ER%) and below 1% (TN < 1ER%); one of the 228 TNBC cases did not have a reported exact ER% score. **B**-**E** Scaled mRNA expression of *PGR, AR, FOXA1* and *FOXC1* for ERpHER2n-Basal tumors compared to TNBC-Basal (TN_B) and TNBC-NonBasal (TN_NB) tumors. **F** Scores of the immune response metagene and tumor infiltrating lymphocyte (TIL) counts of TNBC subtypes. **G** Scaled mRNA expression of *CD274* (*PD-L1*). **H**-**I** Scores of the mitotic checkpoint (proliferation) and steroid response metagenes. **J** Venn diagram of the overlap of differentially expressed genes (DEGs) between Basal vs TNBC-Basal and Basal vs TNBC-NonBasal tumors. **K** UMAP analysis based on FPKM expression data of top 5000 varying genes. **L** Top ten genes with most driver alterations in TNBC-Basal tumors. **M**-**O** Comparison of driver alteration frequencies for *TP53*, *MYC*, and *RB1*. **P** Predicted HRD frequency across subtypes using HRDetect.** Q** Autosomal copy number alteration landscape of ERpHER2n-Basal tumors compared to TNBC-Basal (TN_B) and TNBC-NonBasal (TN_NB). Blue dots mark genes that are significant between ERpHER2n-Basal and TNBC-NonBasal tumors; green dots mark genes that are significant between ERpHER2n-Basal and TNBC-Basal tumors. In panels including boxplots, two-sided p-values were computed using Mann–Whitney's test, top axes report group sizes. Significance annotation: * ≤ 0.05; ** ≤ 0.01; *** ≤ *0.001. Boxplot elements correspond to: (i) center line* = *median, (ii) box limits* = *upper and lower quartiles, (iii) whiskers* = *1.5* × *interquartile range*

We examined the consistency of the Basal-like phenotype across clinical subgroups by comparing the characteristics of ERpHER2n-Basal tumors to those of TNBC tumors, stratified into Basal-like (TNBC-Basal) and non-Basal-like (TNBC-NonBasal) PAM50 subtypes (Fig. 1A). In line with their classification as clinically ER-positive, ERpHER2n-Basal tumors demonstrated significantly higher *ESR1* mRNA expression compared to both TNBC-Basal and TNBC-NonBasal subtypes. However, the overall pattern of *ESR1* expression positioned them closer to the TNBC groups than the ERpHER2n LumA and LumB subtypes (Fig. 4A). Within the context of the discussion around the definition of ER-positivity, we observed that ERpHER2n-Basal tumors still had a statistically significantly higher *ESR1* expression than both TNBC cases with ER staining of 1–9% (also referred to as ER-low; Mann–Whitney U test p ≤ 0.001) and below 1% (Mann–Whitney U test *p* ≤ 0.001). The mRNA expression patterns of *PGR, AR, FOXA1,* and *FOXC1* revealed a consistent similarity between ERpHER2n-Basal and TNBC-Basal tumors (Fig. 4B-E). Immune metagene scores indicated ERpHER2n-Basal tumors to be highly immune infiltrated also when compared to TNBC subgroups. To validate these findings, we utilized available TIL counts from the SCAN-B TNBC cohort, as corresponding data for the ERpHER2n cohort was not available. Our analysis confirmed that differences in immune metagene scores among TNBC subgroups were consistent with their TIL counts, suggesting that ERpHER2n-Basal cases are also likely to exhibit elevated TIL levels (Fig. 4F). Consistently, *PD-L1* (*CD274*) mRNA expression in ERpHER2n-Basal tumors was higher than in TNBC-NonBasal tumors (Fig. 4G). In terms of proliferation metagene scores, as indicated by the mitotic checkpoint metagene, ERpHER2n-Basal, LumB, and TNBC-Basal tumors had higher levels compared to LumA and TNBC-NonBasal tumors (Fig. 4H). Notably, rank scores of the steroid response metagene were reduced in ERpHER2n-Basal tumors compared to TNBC-NonBasal tumors despite the latter being clinically ER-negative (Fig. 4I). Overall, the ERpHER2n-Basal subtype, although clinically ER-positive, shows a striking similarity to the TNBC-Basal group, particularly in its lower dependence on ER-signaling and comparatively higher immune infiltration. It should be noted that due to sample size differences between groups, some observed statistical differences may have minimal effect sizes, indicating limited biological significance.

A differential analysis revealed fewer genes differentially expressed between ERpHER2n-Basal versus TNBC-Basal tumors compared to ERpHER2n-Basal versus TNBC-NonBasal tumors, with only a small gene overlap (Fig. 4J, Additional File 2). UMAP clustering based on the top 5000 most varying genes revealed that TNBC-NonBasal tumors tended to cluster separately from TNBC-Basal and ERpHER2n-Basal tumors (Fig. 4K). The driver alteration landscape of TNBC-Basal tumors also showed a high frequency of *TP53* mutations, similar to ERpHER2n-Basal, followed by mutations in *RB1* and *MYC* (Fig. 4L, M). Both *RB1* and *MYC* mutations were more frequently observed in ERpHER2n-Basal tumors than in any of the non-Basal subtypes (LumA, LumB, TNBC-NonBasal) (Fig. 4N, O). HRDetect classified 71% of TNBC-Basal tumors to be HRD-positive, followed by 44% in ERpHER2n-Basal, with both being significantly more frequent compared to all non-Basal subtypes (Figs. 3F and 4P). On a genome-wide level, ERpHER2n-Basal tumors were similar to TNBC-Basal tumors but differed from TNBC-NonBasal tumors. This was evident from the significant differences in copy number alteration frequencies, with only five genes differing between ERpHER2n-Basal and TNBC-Basal tumors, compared to 393 genes differing between ERpHER2n-Basal and TNBC-NonBasal tumors (Fig. 4Q, Additional File 4).

### DNA methylation landscape indicates a Basal phenotype transcending clinical subgroups

**Fig. 5:**
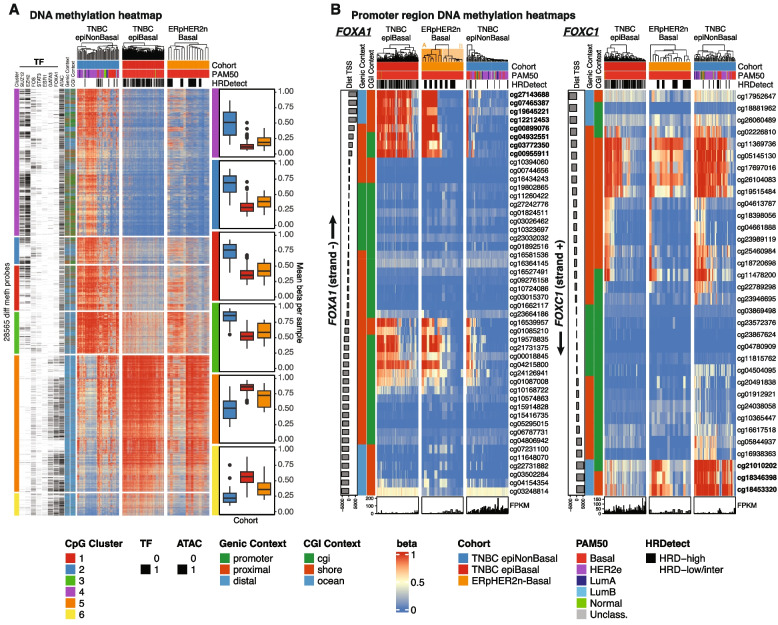
Epigenetic similarity of ERpHER2n-Basal tumors and epiBasal TNBC. **A** DNA methylation heatmap of tumor purity-adjusted beta values for 28565 CpGs of 16 ERpHER2n-Basal tumors and 228 TNBCs epigenetically divided into proposed epiBasal and epiNonBasal epitypes. The plotted CpGs were all found to be statistically differentially methylated between epiBasal TNBC and epiNonBasal TNBC by Aine et al. [13]. Left-hand side annotation tracks show CpG overlap with transcription factor (TF) binding sites, with open chromatin regions in breast cancer (ATAC, since defined by ATAC-seq), and with different genomic regions such as gene promoters and CpG islands (cgi) as defined in Aine et al. Right hand side box plots show mean beta value per sample per CpG row cluster in the tumor groups (indicated by color in the same order as in the heatmap). **B** Promoter region DNA methylation heatmap using tumor purity-adjusted beta values for CpGs +—6000 bp from the transcription start site (TSS) of *FOXA1* (left) and *FOXC1* (right). Strand direction for genes indicated. Lower bar plots show corresponding gene FPKM levels. In panels A and B, sample annotation tracks show cohort, PAM50 subtype, and HRD status. Arrows indicate the direction of transcription for the genes. *All groups are displayed with the same size in the heatmap for visualization purposes despite varying sample numbers. Clustering was performed using Euclidean distance and the”complete” agglomeration method in all panels. CpG IDs in bold indicate differential methylation in direction of expression*

To further analyze the congruence of the ERpHER2n-Basal phenotype with a Basal TNBC phenotype we compared the epigenetic patterns (DNA methylation) of 16 ERpHER2n-Basal tumors to SCAN-B TNBC tumors epigenetically stratified into the epitypes epiBasal (*n* = 172) and epiNonBasal (*n* = 56) based on the study by Aine et al. [13] (Fig. 5). In the study by Aine et al., a set of 28,565 CpGs was reported to be differentially methylated between epiBasal and epiNonBasal TNBC. To test the hypothesis that ERpHER2n-Basal tumors share a DNA methylation profile similar to epiBasal TNBC tumors, we co-analyzed ERpHER2n-Basal and TNBC tumors and displayed DNA methylation values (beta values) for the 28,565 CpGs. As shown in Fig. 5A, ERpHER2n-Basal tumors appear clearly similar to epiBasal TNBC, suggesting a similar epigenetic backbone with respect to these CpGs. As shown in Figs. 2 and 4, two key transcriptional features of ERpHER2n-Basal tumors are downregulation of *FOXA1* and upregulation of *FOXC1* mRNA expression. To analyze if these transcriptional alterations were correlated with epigenetic alterations we created promoter region methylation maps for the SCAN-B tumors, identifying a marked similarity of ERpHER2n-Basal tumors with epiBasal TNBC regarding hypermethylation of shore/CGI CpGs for *FOXA1*, while hypomethylation of shore/CGI CpGs for *FOXC1* (Fig. 5B). Importantly, these results suggest a similar underlying epigenetic regulation of these key transcription factors in Basal tumors irrespective of ER-status. An interesting observation is a greater heterogeneity in promoter region methylation patterns for *FOXA1* within ERpHER2n-Basal tumors compared to epiBasal TNBC. This observation was not predominantly driven by tumor HRD status and corresponded to a difference in PAM50 Basal centroid correlations, difference in mRNA expression of the respective genes (*FOXA1* and *FOXC1*), as well as a difference in *ESR1* expression (Additional File 1: Fig. S2). However, further investigation of this methylation pattern heterogeneity was limited by the relatively low sample number, precluding an in-depth analysis and robust statistical comparisons.

Given the marked similarity between ERpHER2n-Basal tumors and epiBasal TNBC, we next investigated how distinct these patterns were in a general breast cancer context, by analyzing 645 TCGA tumors comprising all clinical subgroups with matched RNA-sequencing and DNA methylation data. Again, we co-analyzed our 16 ERpHER2n-Basal tumors with the TCGA tumors and displayed the data for 14302 of the 28565 CpGs present on the lower resolution Illumina 450 K platform used by the TCGA consortium. As shown in Additional File 1: Fig. S3A, the overall DNA methylation profile of ERpHER2n-Basal tumors closely resembles that of PAM50 classified Basal TCGA tumors, irrespective of ER, PR, and HER2 status, while displaying a markedly different DNA methylation profile compared to luminal tumors. Promoter region methylation heatmaps of *FOXA1* and *FOXC1* in TCGA tumors confirmed a similar methylation pattern for ERpHER2n-Basal tumors and PAM50 Basal TCGA tumors also irrespective of ER, PR, and HER2 status (Additional File 1: Fig. S3B).

### Validation of key molecular findings in the METABRIC cohort

**Fig. 6:**
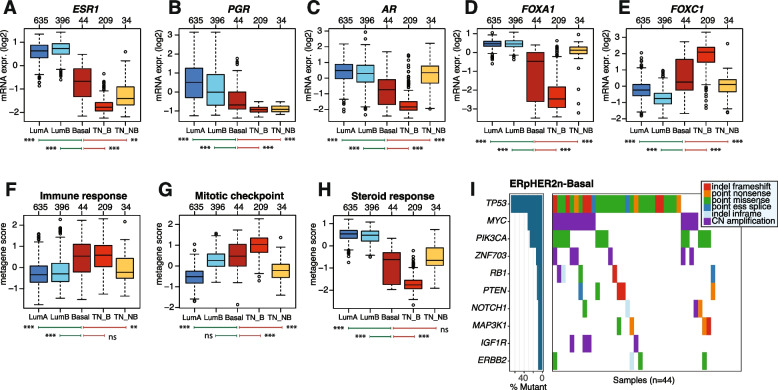
Independent Validation of Molecular Findings in the METABRIC cohort. **A**-**E** Scaled mRNA expression patterns of *ESR1, PGR, AR, FOXA1*, and *FOXC1* across luminal and TNBC tumors stratified by PAM50 subtype (TN_B: TNBC-Basal, TN_NB: TNBC-NonBasal). **F**–**H** Immune response, mitotic checkpoint (proliferation) and steroid response metagene rank scores. **I** Top ten driver gene alteration landscape in ERpHER2n-Basal METABRIC tumors. Significance annotation: * ≤ 0.05; ** ≤ 0.01; *** ≤ *0.001. In panels A-H, two-sided p-values were computed using Mann–Whitney's test, top axes report group sizes. Boxplot elements correspond to: (i) center line* = *median, (ii) box limits* = *upper and lower quartiles, (iii) whiskers* = *1.5* × *interquartile range*

To validate our main observations in the SCAN-B cohort, we investigated key molecular findings, including mRNA expression patterns and driver gene alterations, also in the independent METABRIC cohort (Fig. 1A). Consistent with findings in the SCAN-B cohort, ERpHER2n-Basal METABRIC tumors demonstrated markedly lower expression of *ESR1, PGR, AR*, and *FOXA1*, as well as an elevated expression of *FOXC1*, compared to ERpHER2n LumA and LumB tumors (Fig. 6A-E). ERpHER2n-Basal tumors were associated with higher immune response scores than both LumA and LumB tumors and elevated proliferation similar to LumB tumors (Fig. 6F-G). The lower mRNA expression of ER-associated genes in ERpHER2n-Basal tumors was also reflected in their low rank scores of the steroid response metagene (Fig. 6H). These findings reinforce the identification of a highly proliferative and immune-inflamed phenotype in ERpHER2n-Basal tumors, as well as their diminished steroid hormone responsiveness.

Similar to SCAN-B, the resemblance between the ERpHER2n-Basal and TNBC-Basal subtype in METABRIC was primarily driven by their shared features of high immune infiltration and proliferation activity, as indicated by metagene scores (Fig. 6F-G). Beyond these traits, the separation of ERpHER2n-Basal tumors from TNBC-NonBasal tumors in terms of *FOXA1* and steroid response was a little less distinct in METABRIC compared to SCAN-B (Fig. 6D and H). It should, however, be noted that the used cut-off in ER for defining ER-positivity is most likely lower in METABRIC compared to the ≥ 10% cut-off used in SCAN-B. Analysis of driver gene alterations in METABRIC tumors corroborated our observation of a high frequency of *TP53* mutations, as well as *MYC* amplifications, in ERpHER2n-Basal tumors (Fig. 6I).

## Discussion

In the current study we have comprehensively analyzed the rare, but distinct, subgroup of breast cancer patients with ERpHER2n-Basal tumors. A clinically important feature of this patient subgroup is the poor outcome after adjuvant ET as previously noted [8, 35]. This unfavorable prognosis may reflect reduced estrogen dependence, indicated by a notably lower expression of steroid response associated genes. Activating *ESR1* mutations (e.g., Y537S/D538G) can drive ligand-independent ER signaling, but their absence in the ERpHER2n-Basal tumors is consistent with the observed low expression of steroid response genes [36]. Together, these results suggest that patients with ERpHER2n-Basal tumors could be considered for additional systemic therapies besides ET, stressing the importance of in-depth characterization of this tumor subgroup.

More evidence is emerging that patients with ER-low breast cancers (tumors with 1–9% ER staining) should be considered for clinical trials focused on TNBC [37]. A recent study concluded that ER-low/HER2-negative tumors are part of TNBC and warrant similar treatments, while the benefit of endocrine therapy in these tumors requires prospective evaluation [38]. In this context, a highly important feature of this study is that the main analyses are performed using patients enrolled in the Swedish SCAN-B observational study, for which ER-positivity is defined according to Swedish guidelines (≥ 10% ER score). Thus, SCAN-B ERpHER2n-Basal tumors would be considered ER-positive also in an updated international context, representing a key difference compared to multi-omic tumor cohorts from, e.g., the TCGA or METABRIC studies. This is underscored by our observation of a higher *ESR1* mRNA expression in ERpHER2n-Basal cases compared to TNBC cases with an ER staining of 1–9% (see Fig. 4A). Importantly, the difference in how ER-positivity is defined may also explain the difference in observed ERpHER2n-Basal frequency between the SCAN-B and METABRIC cohorts. As explicit ER percentage from IHC data are not available for METABRIC tumors it was not possible to reclassify these according to Swedish guidelines.

Molecular analyses in the current study clearly demonstrate marked differences between ERpHER2n-Basal and ERpHER2n-LumA/LumB tumors on all investigated levels (DNA, RNA, and epigenetics). Moreover, the comparison of ERpHER2n-Basal tumors to TNBC-Basal and TNBC-NonBasal tumors demonstrates a strong and consistent similarity of the ERpHER2n-Basal group with TNBC-Basal on multiple levels. This included a similar relapse pattern (early relapses), high HRD-frequency, frequent *TP53* mutations and *MYC* copy number alterations, and expression patterns of key transcription factors in breast cancer like the PAM50 genes *FOXA1* and *FOXC1*. The latter was further exemplified also on the DNA methylation level, demonstrating a clear similarity between ERpHER2n-Basal and TNBC-Basal tumors on the global level, but also more specifically for the putative basic gene regulation of *FOXA1* and *FOXC1*. Together, these results provide key evidence of these Basal subgroups being similar, despite the difference in clinical ER classification. Our findings support the hypothesis that basal-like tumors share a cellular origin, likely within the luminal progenitor compartment, as indicated by findings of Lim et al. [39], who identified a strong enrichment of a luminal progenitor molecular signature in these tumors. This idea aligns with the concept of luminal-to-basal plasticity, proposed in a murine lineage-tracing study, which demonstrated that luminal tumors with low ER expression are able to transition towards a basal-like state [40]. Consistent with this, we observed higher *ELF5* mRNA expression in ERpHER2n-Basal and TNBC-Basal tumors, aligning with its role as a key regulator of luminal progenitor differentiation (Additional File 1: Fig. S4). *ELF5* has been shown to promote an ER-negative fate by suppressing *ER* and *FOXA1* expression [41] and to be putatively epigenetically regulated in TNBC [13].

Against the backdrop of poor patient outcomes on adjuvant ET and the need for additional systemic therapies, the biological similarity between TNBC-Basal and ERpHER2n-Basal tumors raises the question of whether patients with ERpHER2n-Basal tumors could benefit from treatment approaches used in TNBC (similar to proposals for the ER-low group [42]). Immune checkpoint inhibitors have recently become clinical routine as part of neoadjuvant therapy in TNBC [9] and are currently being explored for early hormone receptor-positive HER2-negative breast cancer, e.g., in the CheckMate 7FL and KEYNOTE-756 trials [43, 44]. While no outcome data is yet available from subgroup analyses of PAM50 intrinsic molecular subtypes in these studies, the CheckMate 7FL trial demonstrated that adding the anti-PD-1 agent nivolumab to neoadjuvant chemotherapy significantly improved pathological complete response rates in high-risk ERpHER2n breast cancer, particularly in tumors with high stromal TIL levels and/or PD-L1 positivity. They observed a greater benefit in patients with low ER expressing tumors (< 10%), where these features of immunogenicity have previously been shown to be elevated [43, 45]. Although the ERpHER2n-Basal cases in this study were defined by an ER expression ≥ 10%, our study indicates elevated TILs, high *PD-L1* mRNA expression and equivalence of immune activation by mRNA signatures, suggesting that immunotherapy could be of clinical benefit to this subgroup. This is also supported by observations in the GIADA trial that found the combination of a basal-like intrinsic subtype and high TILs could predict pCR after neoadjuvant treatment with chemotherapy, immune checkpoint inhibition, and endocrine therapy in premenopausal women with aggressive HR-positive (ER and/or PR ≥ 10%)/HER2-negative breast cancer [46]. Given evidence that immune activation is associated with response to chemotherapy in TNBC [47], it would be of interest to assess whether immune status similarly influences chemotherapy response in ERpHER2n-Basal tumors, although our current cohort is too small for definitive evaluation.

For patients with high-risk, HER2-negative early breast cancer and germline *BRCA1* or *BRCA2* pathogenic variants, the OlympiA trial demonstrated improved invasive disease-free survival through treatment with the adjuvant PARP inhibitor olaparib [48], recently becoming a part of clinical treatment guidelines [9]. Notably, 44% of our ERpHER2n-Basal cases were predicted as being HRD-positive by HRDetect. Previous studies showed about 21% of HRD predicted TNBC cases to have pathogenic biallelic loss of *BRCA1* or *BRCA2 *[17] and that approximately 81% of these alterations are germline acquired in the general breast cancer population [12]. Given that approximately 60% of *BRCA1/2* carriers are not detected by selective clinical screening of individuals [49], our findings suggest that PAM50 Basal-like classification might be considered as an indication for germline testing in luminal disease, as there would likely be a proportionally high rate of carriers of pathogenic germline *BRCA1* or *BRCA2* variants in these patients. Identifying patients with these alterations has important clinical implications including treatment options, surveillance, prophylactic surgery, and genetic counseling.

The rareness of the ERpHER2n-Basal patient subgroup represents the major limitation of the current study, affecting for instance group sizes and event numbers in survival analyses, as well as available samples for WGS and DNA methylation profiling, including the need to combine ERpHER2n-Basal patients’ tumors with tumors from the external BASIS cohort. Still, even based on a lower number of cases the current study demonstrates considerable molecular similarities between ERpHER2n-Basal and TNBC-Basal tumors. A further consideration is that the METABRIC cohort, which we used as an independent validation dataset, may not fully reflect the general breast cancer population. Nevertheless, the fact that key molecular features identified in the representative SCAN-B cohort were also recapitulated in METABRIC supports the generalizability of our findings. An additional limitation is the lack of in situ confirmation of the elevated immune response pattern observed in ERpHER2n-Basal tumors, which could be further explored for differences in specific immune cell types by e.g. multiplexed IHC or spatial transcriptomics. However, as previously reported, and as illustrated in the TNBC comparison, the specific RNA-sequencing metagene used in this study correlated well with whole slide hematoxylin and eosin (H&E) estimated TIL counts [33], indicating that observed RNA-sequencing patterns would likely correspond to higher TIL counts.

## Conclusions

Taken together, our study demonstrates that ERpHER2n-Basal tumors exhibit strong biological similarities to TNBC-Basal tumors, despite the difference in clinical ER classification. These similarities raise important questions regarding optimal treatment strategies for this patient subgroup. Our findings suggest that incorporating immune checkpoint inhibitors or PARP inhibitors into treatment considerations could be of clinical relevance, particularly given the demonstrated benefit of these therapies in TNBC and high-risk HRD-positive breast cancers.

## Supplementary Information

Additional file 1. Supplementary Methods and Figures. This PDF file contains the supplementary methods and figures.

Additional file 2. Differential gene expression analysis results. This Excel file provides the results of the differential gene expression analysis of ERpHER2n Basal-like cases vs. ERpHER2n and TNBC subgroupsand the core set of differentially expressed genes.

Additional file 3. SCAN-B ERpHER2n Basal-like processed somatic WGS data. This Excel file provides the processed somatic WGS data for 16 Basal-like tumors.

Additional file 4. ERpHER2n Basal-like copy number data and alteration frequencies. This Excel file provides gene-level copy number gain and loss frequencies per subgroup. It also provides the results of statistical tests comparing these frequencies in ERpHER2n Basal-like cases to other subgroups and the genes found to be significantly differing in these comparisons.

Additional file 5. ERpHER2n Basal-like clinical annotations. This Excel file provides the clinical annotations of SCAN-B ERpHER2n Basal-like cases included in the study.

## Data Availability

The previously reported DNA methylation data for SCAN-B ERpHER2n-Basal tumors used in this study are available from the study by Hohmann et al. [16] through the Gene Expression Omnibus database under accession code GSE278586 [https://www.ncbi.nlm.nih.gov/geo/query/acc.cgi?acc=GSE278586] (accession IDs listed in Additional File 5). The previously reported DNA methylation data for SCAN-B TNBC tumors used in this study are available from the study by Aine et al. [13] through the Gene Expression Omnibus database under accession code GSE148748 [https://www.ncbi.nlm.nih.gov/geo/query/acc.cgi?acc=GSE148748] and GSE148906 [https://www.ncbi.nlm.nih.gov/geo/query/acc.cgi?acc=GSE148906]. All SCAN-B RNA-sequencing data used in this study have been previously reported by Staaf et al. [5] and are available from [https://data.mendeley.com/datasets/yzxtxn4nmd/3]. The generated raw whole genome sequencing data for SCAN-B cases are protected and are not available due to data privacy laws and patient consent. The processed somatic whole genome sequence tumor data supporting reproduction are available in Additional File 3. The previously reported TCGA data used in this study were generated by the TCGA breast tumor study [14] and are available through the GDC data portal [https://portal.gdc.cancer.gov]. The previously reported METABRIC data used in this study are available from the study by Curtis et al. [11] through the cBioPortal website (https://www.cbioportal.org/). Raw data for the BASIS cohort used in this study are available from the study by Nik-Zainal et al. [12] through the EGA repository [https://ega-archive.org/studies/EGAS00001001178]. The scripts for all analyses that were performed as part of this study are freely accessible at: https://github.com/StaafLab/ERpHER2n-Basal_study [50].

## Abbreviations

BC: Breast Cancer
ER: Estrogen Receptor
PR: Progesterone Receptor
HER2: Human Epidermal Growth Factor Receptor 2
ERpHER2n: Estrogen Receptor-positive, HER2-negative
TNBC: Triple-Negative Breast Cancer
LumA: Luminal A
LumB: Luminal B
SCAN-B: Sweden Cancerome Analysis Network-Breast
METABRIC: Molecular Taxonomy of Breast Cancer International Consortium
TCGA: The Cancer Genome Atlas
DRFI: Distant Recurrence-Free Interval
OS: Overall Survival
ET: Endocrine Therapy
CT: Chemotherapy
IHC: Immunohistochemistry
FISH: Fluorescence In Situ Hybridization
WGS: Whole Genome Sequencing
FPKM: Fragments Per Kilobase of transcript per Million mapped reads
RS: Recurrence Score
ROR: Risk of Recurrence
LN: Lymph Node
NHG: Nottingham Histologic Grade
TIL: Tumor-Infiltrating Lymphocyte
DEG: Differentially Expressed Gene
GO: Gene Ontology
HR: Hazard Ratio
CI: Confidence Interval
HRD: Homologous Recombination Deficiency
SBS: Single Base Substitution
RS: Rearrangement Signature
SNV: Single Nucleotide Variant
GEO: Gene Expression Omnibus
