# Supplementary material for: Molecular profiling of the Basal-like intrinsic molecular subtype in primary ER-positive HER2-negative breast cancer
Source: Genome Med. 2025 Dec 1;17:146. doi: 10.1186/s13073-025-01576-9 (PMC12667147; doi:10.1186/s13073-025-01576-9)
Supplement: Supplementary file 1 — Additional file 1. Supplementary Methods and Figures. This PDF file contains the supplementary methods and figures. [file 13073_2025_1576_MOESM1_ESM.docx]

**SUPPLEMENTARY METHODS AND FIGURES**

**1. Supplementary Methods**

**Analysis of clinicopathological and molecular subtype variables**

Fisher’s exact tests were used to evaluate differences in tumor grades, lymph node status, and PR status. Mann-Whitney U tests were applied to assess the clinicopathological variables age and tumor size. SCAN-B samples were classified into PAM50 subtypes using the extended Nearest Centroid (NCN) method, as implemented by Staaf et al. This approach extends traditional nearest centroid classification by incorporating multiple reference sets for normalization instead of relying on a single set (see Staaf et al.(1) for further details on NCN). The final PAM50 subtype for each sample was assigned based on a majority vote across 100 classification iterations, while the centroid correlations for PAM50 subtypes were averaged over all 100 iterations in the NCN process. 21-gene Recurrence Scores for LumA, LumB and Basal ERpHER2n tumors were calculated based on RNA-sequencing FPKM data using the Genefu R package (v2.36.0) and classified as either high, intermediate or low-risk cases(2). The ROR scores and risk groups were previously calculated by Staaf et al.(1) and provided in Supplementary Table 1 of that study. Specifically, we utilized the “NCN.ROR.risk.cat” column to obtain ROR risk groups. The ROR score was derived from the “NCN.ROR.asT0 column” for tumors ≤20 mm, and from the “NCN.ROR.asT1” column for tumors >20 mm. Tumor size information was also provided as column “Size.mm” in the supplementary file. Statistical significance of differences in 21-gene Recurrence Scores, ROR scores, and risk classifications were assessed using Mann-Whitney U and Fisher’s exact tests, respectively.

The median outcome measures in censored patients were equal to 5.3 years (DRFI, ET, SCAN-B), 5.5 years (DRFI, CT+ET, SCAN-B), 7.1 years (OS, ET, SCAN-B), and 6.7 years (OS, CT+ET, SCAN-B). The SCAN-B release forming the main cohort for this study includes patients enrolled between 2010 and 2018. SCAN-B clinical review data from 2010 to 2014 indicate that ERpHER2n patients receiving chemotherapy were treated with FEC and Docetaxel, while ET patients received tamoxifen and/or aromatase inhibitors. However, detailed treatment data for SCAN-B patients enrolled between 2015 and 2018, as well as for those in the METABRIC and BASIS cohorts, were unavailable.

**Gene expression analyses**

For all gene expression analyses, preprocessing of the SCAN-B cohort data involved log2 transformation of FPKM gene expression values with a +1 offset, followed by gene-wise scaling using a z-transformation. For comparisons within the ERpHER2n group, scaling was performed over the ER-positive SCAN-B samples. In contrast, for analyses including TNBC groups, gene expression data was scaled across the entire SCAN-B cohort. Deposited METABRIC data was already preprocessed (including z-transformation) and therefore directly used. Gene expression scores for reported biological metagenes related to breast cancer biology were calculated for each sample as the average of all associated gene expression values(3). Differentially expressed genes were identified by two-sided Mann-Whitney U tests in combination with false-discovery rate (FDR) to correct for multiple testing by employing the *p.adjust* function of the *stats* R package (v3.6.2). Genes with an adjusted p-value ≤0.05 were considered as differentially expressed. To investigate potential subtype-associated gene expression patterns, unsupervised UMAP analysis based on the preprocessed FPKM values was conducted using the umap R package (v0.2.10.0).

**Mutational Signature analysis**

Mutational enrichment analysis was conducted to identify potential mutational processes and driver alterations specific to the ERpHER2n-Basal subtype. In the SCAN-B cohort, analyses were based on whole-genome sequencing (WGS) data from Basal tumors (n=16) and supplemented with WGS data from the BASIS cohort, using PAM50 subtype classifications from the original BASIS study (LumA n=73; LumB n=105). Preprocessed and filtered BASIS mutational data were obtained from a publicly available repository linked to the original study.

For each SCAN-B patient, tumor samples collected during surgery, along with matched blood DNA, were sequenced at Novogene (UK) using 150 bp paired-end sequencing on an Illumina NovaSeq platform. Sequencing aimed to generate 120–150 GB of data, resulting in tumor coverage between 26–50× (median 34×) after duplicate removal and final filtering. BAM files were aligned to the human reference genome (GRCh38) using dockstore-cgpmap v3.2.0, which implements bwa mem 0.7.17-r1188 (<https://quay.io/repository/wtsicgp/dockstore-cgpmap>).

Mutation calling of WGS data for SCAN-B tumors was performed as described previously(4). The mutation detection workflow was containerized within dockstore-cgpwgs v2.1.1 (<https://quay.io/repository/wtsicgp/dockstore-cgpwgs>) and incorporated several tools: Caveman 1.13.15 for somatic substitution calling (CaVEMan: <http://cancerit.github.io/CaVEMan/>), Pindel 3.2.0 for identifying small somatic insertions and deletions (<http://cancerit.github.io/cgpPindel/>), and BRASS for detecting structural rearrangements (BReakpoint AnalySiS; <https://github.com/cancerit/BRASS>). Tumor ploidy and purity estimates, used as input for Caveman, were provided by ASCAT 4.2.1 (<https://github.com/cancerit/ascatNgs>).

Additional filtering criteria were applied to refine variant calls: single base substitutions (SBSs) were retained if they met PASS criteria, CLPM=0.00, and ASMD≥140; small indels were included if they had QUAL≥250 and REP<10; structural variants were filtered based on a BRASS assembly score >0, ensuring successful de novo local assembly using Velvet for precise breakpoint identification.

Mutational signatures were identified using the Fit Multi-Step (FitMS) approach(5) (<https://github.com/Nik-Zainal-Group/signature.tools.lib>) with the following parameters. For substitution signatures, reference SBS signatures previously detected in breast cancer were used alongside high-confidence rare signatures from various tissues. A fixed threshold was set at 5% of total mutations contributing to a signature before assignment, with an error reduction of 20%. For structural rearrangement signatures, previously established breast cancer reference signatures were fitted to samples with more than 25 total rearrangements. Additionally, mutational signatures were assigned only if at least five variants contributed and if they accounted for at least 5% of the total rearrangements. The resulting signatures were used as input to HRDetect(6).

**Driver mutation analysis**

Somatic mutations were annotated using Ensembl v91 with VAGrENT (<https://github.com/cancerit/VAGrENT>). Non-synonymous point mutations and small indels were evaluated for potential driver status by comparing them to genes listed in the Cancer Gene Census (<https://cancer.sanger.ac.uk/census>) and those previously identified as breast cancer driver genes(4). A mutation was considered a potential driver if it was recurrently observed in the COSMIC database or classified as pathogenic/likely pathogenic in cancer according to ClinVar. Additionally, mutations in tumor suppressor genes listed in the Cancer Gene Census (excluding those primarily altered through gene fusions) were classified as potential drivers if they were predicted to cause premature truncation, such as nonsense, essential splice site, or frameshift mutations. Loss of heterozygosity (LOH) in tumor suppressor genes was assessed using ASCAT-derived copy number data for the corresponding segment.

In the SCAN-B/BASIS cohort, mutational and rearrangement signatures, as well as overall tumor mutational burden, were compared across PAM50 subtypes, with statistical significance evaluated using Mann-Whitney U tests. For the METABRIC dataset, mutational calls were originally generated through a targeted NGS DNA-based panel(7, 8) and obtained as preprocessed data. The mutational landscape of SBSs, indels, and copy number driver genes within the Basal subtype was visualized using waterfall plots generated with the GenVisR package (v1.34.0)(9). Differences in target gene mutational frequencies were statistically assessed using Fisher’s exact tests.

**DNA methylation analyses**

Beta values, representing the level of methylation, were computed in a sample-by-sample context using the minfi R package (v1.44) function *preprocessNoob()* and Infinium probe normalized using the approach described by Holm et al.(10). To account for tumor purity, beta values were adjusted using the reference_based_beta_correction() function from the PureBeta pipeline (see (11) for details) using WGS-based tumor purity estimates (ASCAT values) as input values. We applied the function with default parameters (including the refitting option set to false) and used reference CpG models previously established by Aine et al. (Nature Communication, Accepted 2025).

**Copy number alteration analyses**

Copy number analyses for SCAN-B cases (n=16 Basal) were conducted using copy number estimates derived from WGS data. Basal copy number profiles from SCAN-B were compared to those of ER-positive HER2-negative LumA (n=73) and LumB (n=105) tumors from the BASIS cohort. WGS data processing for copy number analysis was performed using a modified version of ASCAT v3.1.2, with reference files available at <https://github.com/VanLoo-lab/ascat/tree/master/ReferenceFiles/WGS>. Modifications to the ASCAT algorithm are detailed at <https://github.com/nnordborg/ascat/tree/scanb> ("Changes in the SCANB-fork"). The baseline parameters used included imbalance.test=bimodality_coefficient and tau=0.4. Copy number gains and losses were determined based on tumor ploidy, as described by Staaf et al. (Staaf et al., 2019).

To compare genomic alterations between SCAN-B and BASIS, ASCAT segments were mapped to gene coordinates from the respective genome builds (hg19 for BASIS and hg38 for SCAN-B) using the GenomicRanges R package (v1.50.2) (Lawrence et al., 2013). The total number of copy number alterations per tumor was summed, and the fraction of altered genomes was compared across PAM50 subtypes using Mann-Whitney U tests. Differences in gene-specific copy number gains or losses between subtypes were evaluated using Fisher’s exact tests, with false discovery rate (FDR) correction applied for multiple testing. For genes with significant differences in alteration frequency, absolute frequency differences were calculated and visualized using boxplots.

**References**

1. Staaf J, Häkkinen J, Hegardt C, Saal LH, Kimbung S, Hedenfalk I, et al. RNA sequencing-based single sample predictors of molecular subtype and risk of recurrence for clinical assessment of early-stage breast cancer. NPJ Breast Cancer. 2022;8(1):94.

2. Gendoo DM, Ratanasirigulchai N, Schröder MS, Paré L, Parker JS, Prat A, Haibe-Kains B. Genefu: an R/Bioconductor package for computation of gene expression-based signatures in breast cancer. Bioinformatics. 2016;32(7):1097-9.

3. Fredlund E, Staaf J, Rantala JK, Kallioniemi O, Borg A, Ringnér M. The gene expression landscape of breast cancer is shaped by tumor protein p53 status and epithelial-mesenchymal transition. Breast Cancer Res. 2012;14(4):R113-R.

4. Nik-Zainal S, Davies H, Staaf J, Ramakrishna M, Glodzik D, Zou X, et al. Landscape of somatic mutations in 560 breast cancer whole-genome sequences. Nature. 2016;534(7605):47-54.

5. Degasperi A, Zou X, Amarante TD, Martinez-Martinez A, Koh GCC, Dias JML, et al. Substitution mutational signatures in whole-genome-sequenced cancers in the UK population. Science. 2022;376(6591).

6. Davies H, Glodzik D, Morganella S, Yates LR, Staaf J, Zou X, et al. HRDetect is a predictor of BRCA1 and BRCA2 deficiency based on mutational signatures. Nat Med. 2017;23(4):517-25.

7. Curtis C, Shah SP, Chin SF, Turashvili G, Rueda OM, Dunning MJ, et al. The genomic and transcriptomic architecture of 2,000 breast tumours reveals novel subgroups. Nature. 2012;486(7403):346-52.

8. Pereira B, Chin SF, Rueda OM, Vollan HK, Provenzano E, Bardwell HA, et al. The somatic mutation profiles of 2,433 breast cancers refines their genomic and transcriptomic landscapes. Nat Commun. 2016;7:11479.

9. Skidmore ZL, Wagner AH, Lesurf R, Campbell KM, Kunisaki J, Griffith OL, Griffith M. GenVisR: Genomic Visualizations in R. Bioinformatics. 2016;32(19):3012-4.

10. Holm K, Staaf J, Lauss M, Aine M, Lindgren D, Bendahl PO, et al. An integrated genomics analysis of epigenetic subtypes in human breast tumors links DNA methylation patterns to chromatin states in normal mammary cells. Breast Cancer Res. 2016;18(1):27.

11. Sasiain I, Nacer DF, Aine M, Veerla S, Staaf J. Tumor purity estimated from bulk DNA methylation can be used for adjusting beta values of individual samples to better reflect tumor biology. NAR Genom Bioinform. 2024;6(4):lqae146.

**2. Supplementary Figures**

**
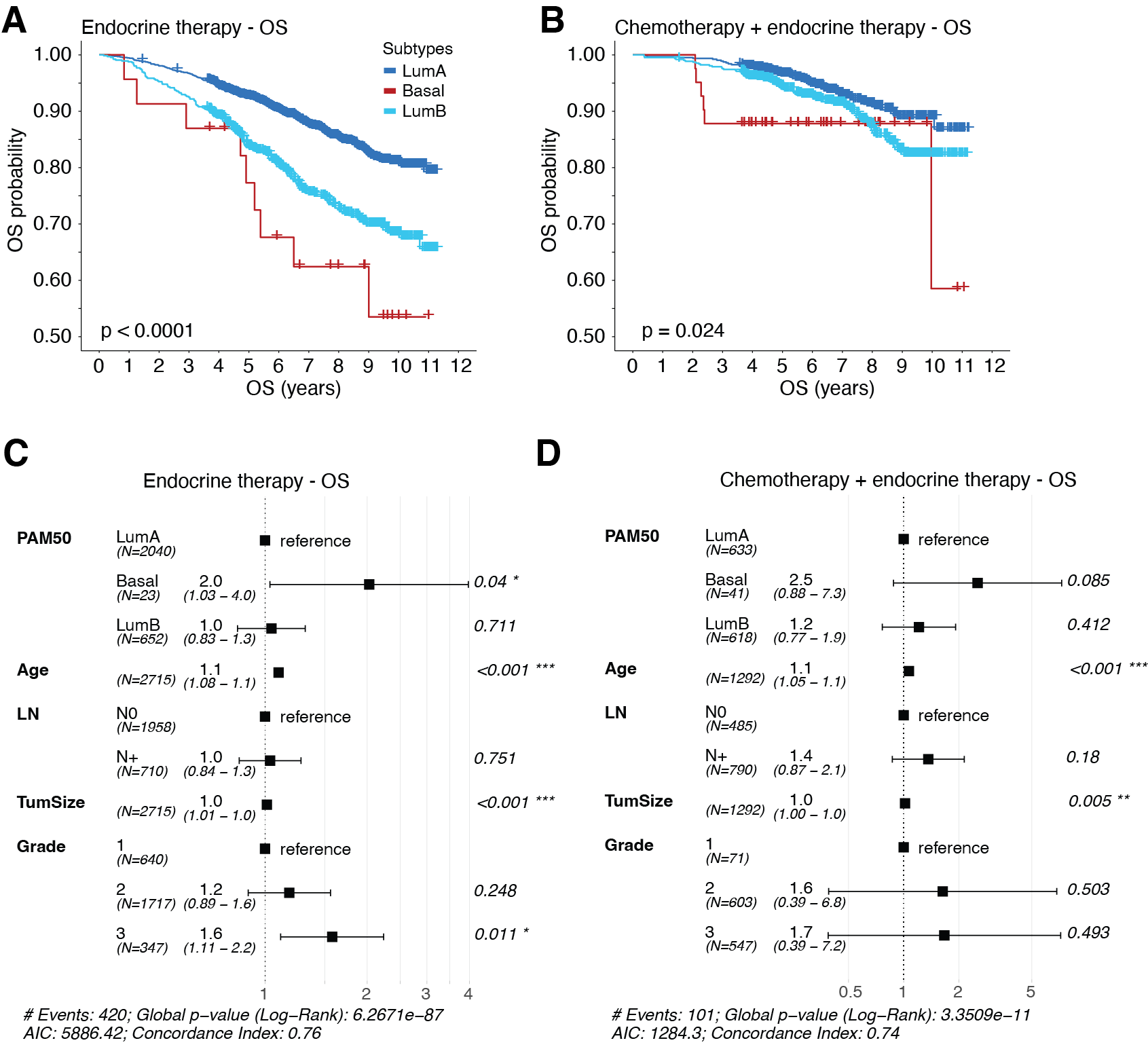
**

**Fig. S1: Survival analyses in ERpHER2n SCAN-B patients with overall survival (OS) as clinical endpoint.** A Kaplan-Meier curve of patients treated with endocrine therapy (ET). B Kaplan-Meier curve of patients treated with chemotherapy and endocrine therapy (CT+ET). C Multivariate Cox regression HRs with 95% CIs for patients treated with ET. D Multivariate Cox regression HRs with 95% CIs for patients treated with CT+ET. Differences in Kaplan-Meier curve p-values calculated using log-rank tests.

**
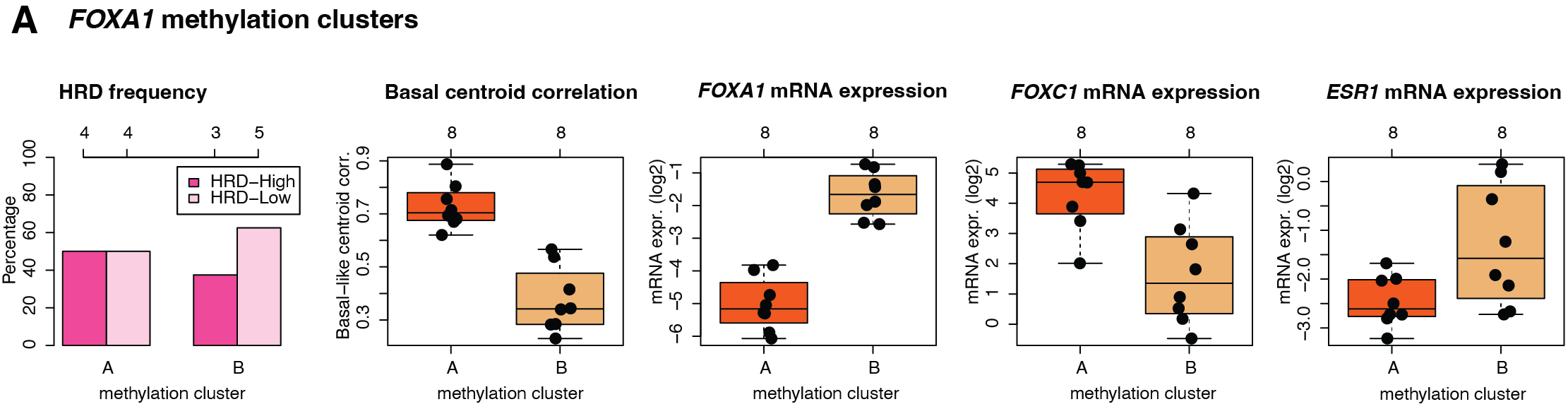
**

**Fig. S2: Comparison of methylation clusters in FOXA1.** A Boxplots comparing clusters defined based on promoter region DNA methylation patterns of FOXA1 in relation to HRD status, PAM50 Basal centroid correlation and mRNA expression of ESR1, FOXA1, and FOXC1. All samples included can be seen in the main test Figure 5B. Top axis labels represent sample sizes.

**
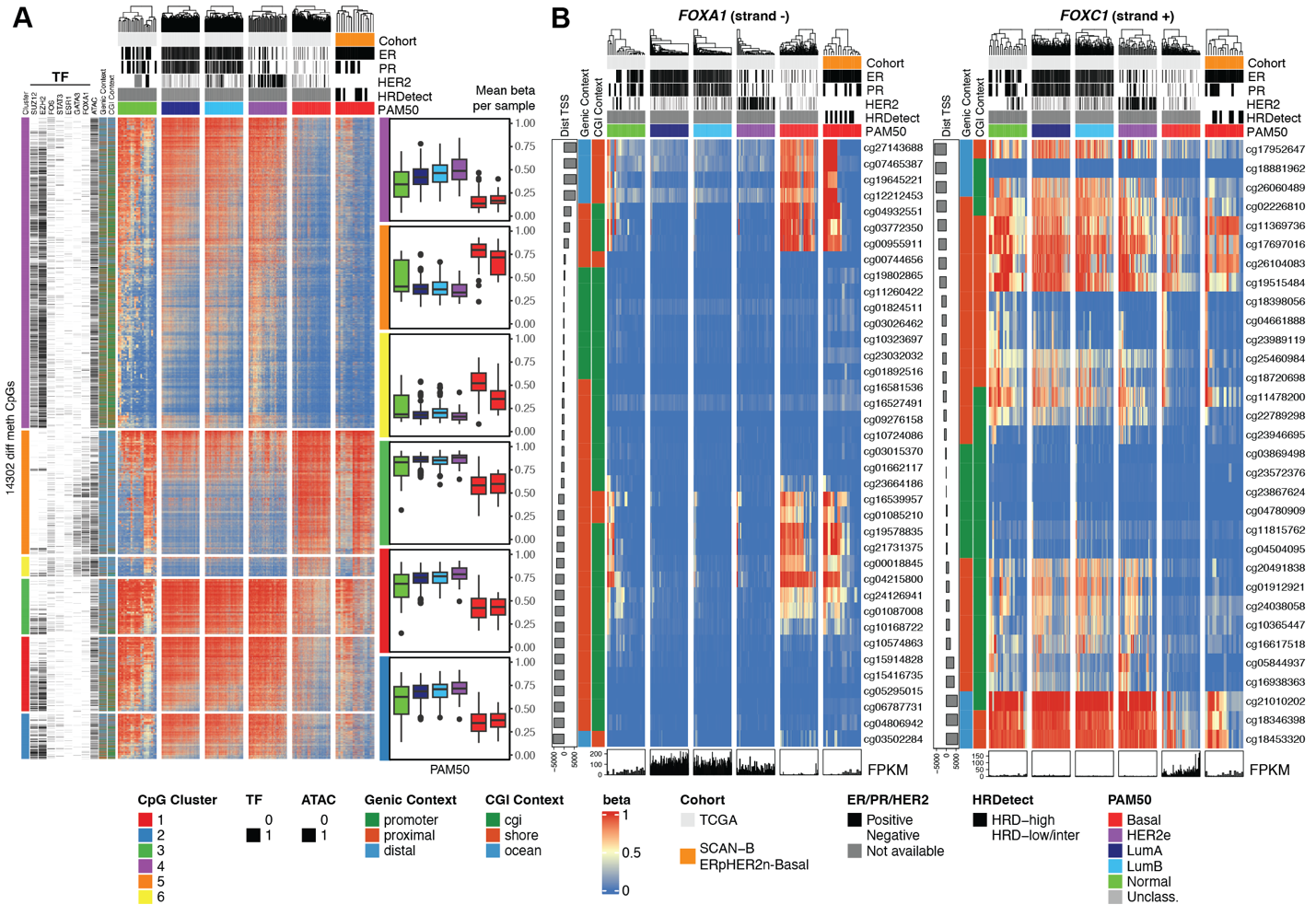
**

**Fig. S3: Comparison of DNA Methylation Profiles between ERpHER2n-Basal tumors and tumors of the TCGA Cohort.** A Heatmap of DNA methylation profiles for 14,302 CpGs from 645 TCGA tumors, including all clinical subgroups, merged with 16 SCAN-B ERpHER2n-Basal tumors. The CpGs represent those present on the Illumina 450K platform. B Promoter region DNA methylation heatmap for CpGs +- 5000 bp from the transcription start site of FOXA1 (left) and FOXC1 (right). Strand direction for genes indicated. Lower bar plots show corresponding gene FPKM levels. In panels A and B, sample annotation tracks show cohort, ER/PR/HER2 status, PAM50 subtype, and HRD status (by HRDetect).

**
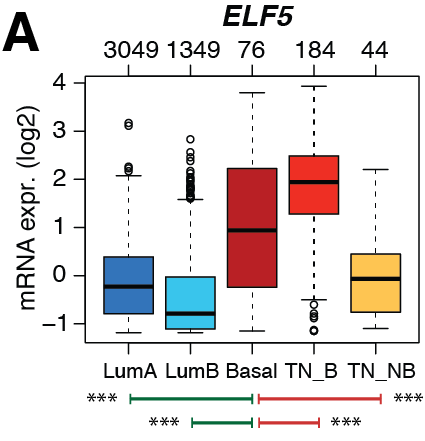
**

**Fig. S4: ELF5 mRNA expression in ERpHER2n and TNBC subtypes.** TNBC-Basal (TN_B); TNBC-NonBasal (TN_NB).
